# Position a Remedy for Stertor

**Published:** 1861-02

**Authors:** 


*Position a Remedy for Stertor.*—Mr. Bowels, after repeated trial, assures the profession that stertor in apoplexy, etc., is immediately relieved by turning the patient well on his side, so that the paralyzed tongue and velum palati will fall forward, and the mucus drain away. Very soon, also, the phenomenon of partial suffocation accompanying the stertor, will disappear. —*Cin. Lancet & Observer.*

